# Surgery of the Heart1Read before the surgical section, American Medical Association, Saratoga, N.Y., June 10-13, 1902.

**Published:** 1902-11

**Authors:** 


### ABSTRACTS.

### Surgery of the Heart.[1]

B. MERRILL RICKETTS, of Cincinnati, O., says injuries and surgery of the heart have, until recently, been classed as anomalies. This one fact shows how little confidence there has been in successfully dealing with the heart surgically. At one time simple needle puncture of the heart was thought to always result in instant death. Experimental physiology and surgery shows what can be done and how to do it. It is the basis upon which the heart surgery especially has been placed.

Twenty-five dogs were used in the experimentations. Penetrating and non-penetrating wounds of the heart were made and closed with sutures of different material. Interrupted silk sutures were found to be the best. No especial aseptic precautions were taken as all pathologic conditions were desired. The pericardium may be entirely removed without death resulting. Either one of the coronary arteries may be ligated at its base without producing death. In a certain class of cases it is best to suture the pericardium to the chest wall that drainage may be perfect.

It is ideal to suture during systole, but one will be satisfied to secure perfect suturing in systole or diastole. Even though the auricular is thinner than the ventricular wall it may be sutured with equal success. Owing to this difference in thickness the percentage of penetrating wounds of the auricles is much greater than those of the ventricles. Knotting of the sutures should be firmly secured, otherwise they may become untied by the constant action of the heart.

The sutures should pass through the bottom of the wound when non-penetrating and through the endocardium when penetrating. If not in the latter, the wound may become enlarged from within. Sutures should not be made tight enough to cut the heart tissue. The mortality is less in wounds of the right than those of the left auricle and ventricle. Bleeding is more severe in wounds from sharp instruments than when due to bullets.

CONCLUSIONS.

1. Injuries and diseases of the heart have resisted surgery longer than almost any of the tissues or organs of the human body.

---

[1] Read before the surgical section, American Medical Association, Saratoga, N.Y., June 10-13, 1902.

2. They, however, no longer offer such resistance but find themselves subject to attack by the same surgical principles as other parts of the body.

3. Experimental surgery teaches one to reason from animal to man.

4. Aneurism, foreign bodies, ossification, together with abscess, syphilis and gangrene possess features which will have a great bearing upon, and will greatly influence the future surgical work of the heart.

5. The application of surgical principles in certain cases of aneurism of the heart will, no doubt, be accomplished by suture, electrolysis, or the injection of gelatine or something of a similar character.

6. The removal of a certain class of foreign bodies, whether they have formed within or have entered from without, should, and no doubt will, be accomplished.

7. That a cardiac abscess should be incised and drained there can be no doubt.

8. Tumors of a pedunculated character on the external surface of the heart can and should be removed.

9. Pedunculated tumors within the cardiac chambers can also be successfully removed.

10. Parasitic cysts (animal or vegetable) when upon the external surface of the heart or in its wall should be incised and drained.

11. Mitral stenosis, hypertrophy and dilatation of the heart will sooner or later find complete or partial relief within the domain of surgery.

12. Injuries involving the myocardium are subject to the same surgical principles as injuries to other important organs of the human body.

13. Lacerated or incised penetrating or non-penetrating wounds of the heart should be sutured.

14. Suturing or any other surgical procedure should not be discontinued because the heart should cease to pulsate. The work can and should be completed within a much shorter time on a quiescent heart.

15. All means should be resorted to, while the suturing of the myocardium is being completed, to reëstablish the heart's action.

16. Drainage of the pericardial sac is necessary in many cases of injury of the heart.

17. Exploratory incision of the pericardial cavity and its contents has been shown by both experimental research and operations upon the living human body to be exceedingly rational, valuable and justifiable.

18. Exploration of the heart itself by puncturing it with a needle or knife to locate a foreign body or to detect pathologic conditions within the myocardium or its chambers, will at no far distant day be found useful, necessary and recognised as an accepted surgical procedure.

19. Why should these conclusions be fallacious when it has already been shown that nine of the twenty-seven cases of heart wounds treated by suture have recovered?

